# Retrospective analysis and prospective validation of an AI-based software for intracranial haemorrhage detection at a high-volume trauma centre

**DOI:** 10.1038/s41598-022-24504-y

**Published:** 2022-11-18

**Authors:** Adil Zia, Calvin Fletcher, Shalini Bigwood, Prasanna Ratnakanthan, Jarrel Seah, Robin Lee, Helen Kavnoudias, Meng Law

**Affiliations:** 1 Department of Radiology, Alfred Health, Melbourne, VIC Australia; 2 Department of Neuroscience, Monash University, Melbourne, VIC Australia

**Keywords:** Brain imaging, Machine learning, Computed tomography

## Abstract

Rapid detection of intracranial haemorrhage (ICH) is crucial for assessing patients with neurological symptoms. Prioritising these urgent scans for reporting presents a challenge for radiologists. Artificial intelligence (AI) offers a solution to enable radiologists to triage urgent scans and reduce reporting errors. This study aims to evaluate the accuracy of an ICH-detection AI software and whether it benefits a high-volume trauma centre in terms of triage and reducing diagnostic errors. A peer review of head CT scans performed prior to the implementation of the AI was conducted to identify the department’s current miss-rate. Once implemented, the AI software was validated using CT scans performed over one month, and was reviewed by a neuroradiologist. The turn-around-time was calculated as the time taken from scan completion to report finalisation. 2916 head CT scans and reports were reviewed as part of the audit. The AI software flagged 20 cases that were negative-by-report. Two of these were true-misses that had no follow-up imaging. Both patients were followed up and exhibited no long-term neurological sequelae. For ICH-positive scans, there was an increase in TAT in the total sample (35.6%), and a statistically insignificant decrease in TAT in the emergency (− 5.1%) and outpatient (− 14.2%) cohorts. The AI software was tested on a sample of real-world data from a high-volume Australian centre. The diagnostic accuracy was comparable to that reported in literature. The study demonstrated the institution’s low miss-rate and short reporting time, therefore any improvements from the use of AI would be marginal and challenging to measure.

## Introduction

Brain computed tomography (CT) imaging is essential in detecting intracranial haemorrhage (ICH), a condition where timely diagnosis and management is essential^1^. In Australia, the use of diagnostic imaging has increased rapidly, with a study finding that the number of billed CT scans rose by over 300% from 1993/94 to 2012/13^2^. This amplified workload on Australian radiologists is projected to increase with time, due in part to an ageing population, increasing complexity of radiological scans, and increased supply of other health professionals, especially subspecialists^3^.

Artificial intelligence (AI) decision support systems have become prolific in recent years, aiming to (1) assist in triage of urgent cases via flags and widgets, as well as insertion of secondary captures into the picture archiving and communication system (PACS) workstation and (2) act as an automated second-read quality assurance checker to avoid diagnostic misses by human radiologists^4–6^. Aidoc, a widely used FDA-approved commercial AI decision support system^7^ for the detection of ICH has been validated previously to have a sensitivity of 88.7 to 96.2% and a specificity of 92.3 to 99.0%^4,7–9^. Recent studies have noted that Aidoc for ICH can decrease scan view delay by 10% in inpatients^10^, decrease turn-around-time for flagged cases^5,11^ and reduce length of stay for Emergency Department (ED) patients who did not have ICH^5^.

Deep learning models, such as Aidoc, are known to have inconsistencies in performance when used on different clinical sites^4,12^. Hence, there remains uncertainty in an Australian context as to whether Aidoc can maintain performance, improve radiologist efficiency, and detect clinically significant abnormalities that a clinical radiologist may have missed.

The present study aims to first apply the Aidoc ICH detection algorithm retrospectively, on ICH-negative studies, to assess the pre-implementation radiologist’s miss-rate, and to determine whether these were clinically significant to patient care. Second, to evaluate the prospective diagnostic accuracy of Aidoc’s ICH detection algorithm at the same tertiary hospital, in terms of diagnostic accuracy and changes in turn-around-time (TAT).

## Methods

Approval for this single-centre retrospective study was provided by The Alfred Hospital Human Research Ethics Committee (Local Project Number 519/19). The study was considered low-risk and the requirement for patient informed consent was waived as per the Alfred Hospital Human Research Ethics Committee. All methods were conducted in adherence to ICH/GCP requirements.

The institution has a total of five CT scanners that have ≥ 64 detectors; two Aquilion One (Canon Medical Systems), two Revolution (GE Healthcare), and one Optima (GE Healthcare). All non-contrast CT scans (NCCT) of the head were performed in an axial plane with 0.625 mm slice thickness. All images are routinely assessed to ensure consistent signal-to-noise ratios between scanners within the department. The CT scans were interpreted by either consultant neuroradiologists, consultant radiologists, or senior radiology registrars.

All head CT scans were analysed using a proprietary TGA-approved AI software, based on a convolutional neural network (CNN) architecture (Aidoc “BriefCase” v.1.3. Tel Aviv, Israel). The ICH triage and notification module was loaned to the institution for a 6-month trial period.

**Figure 1:**
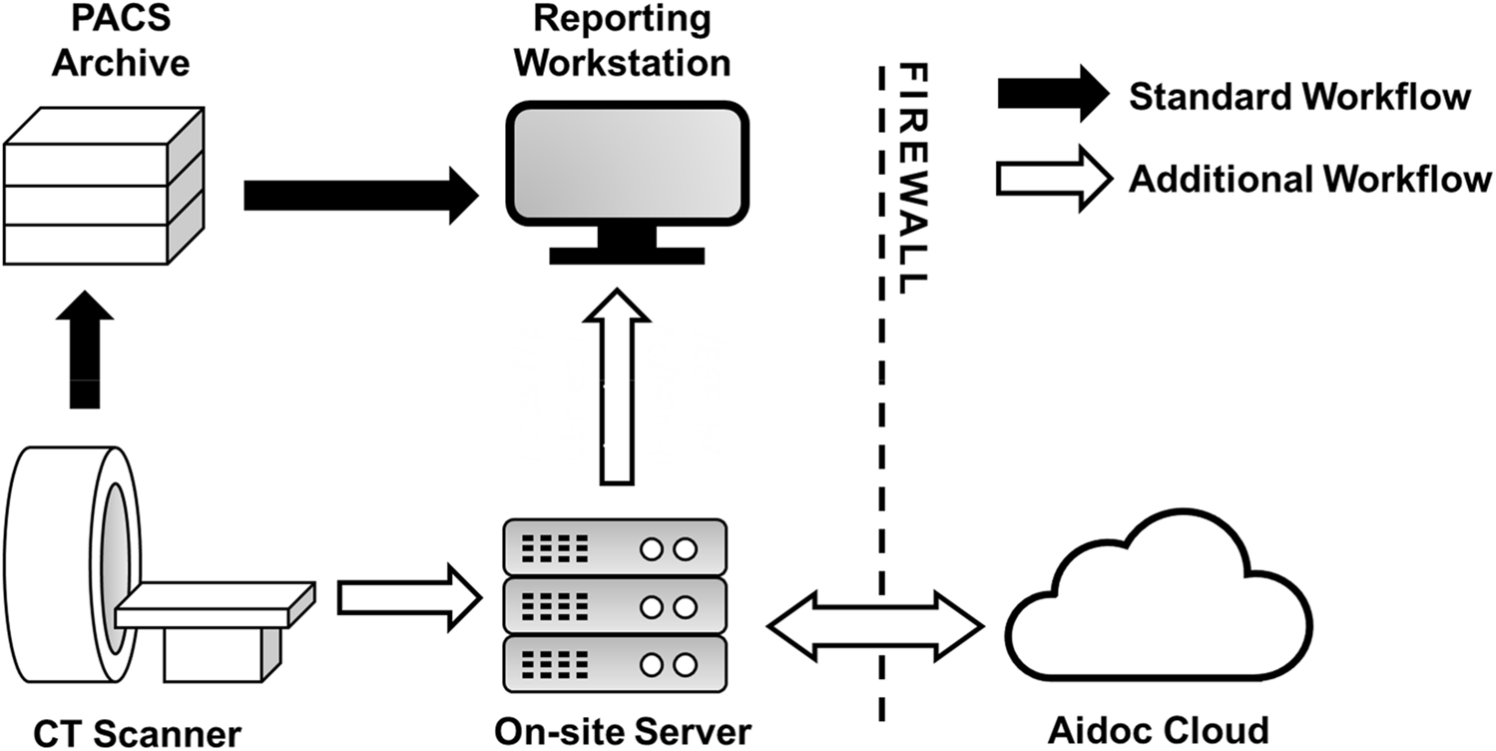
The integration of Aidoc in the institution’s current imaging and reporting systems. The integration allows for real-time analysis of non-contrast head CT scans. All data was de-identified before being transferred beyond the hospital firewall for cloud-based image analysis.

CT scans that included motion artefacts, severe metal artefacts or an inadequate field of view were deemed technically inadequate and were excluded from analyses. DICOM files were de-identified per the HIPAA safe harbor de-identification protocol before being transferred beyond the institution’s firewall for cloud-based analysis. All 18 fields defined by the protocol were removed, and a unique study ID was assigned to each exam. Imaging data was re-identified when returned to the on-site virtual machine, which routed the flagged studies to the desktop-based widget on several radiologist workstations, shown in Fig. 1.

### Retrospective Peer Review

All clinically-acquired NCCT scans of the head were collected from September 2019 to October 2019—prior to the implementation of the ICH-detection into the radiologists’ workflow.

#### Report classification and image analysis

To classify each case as positive or negative for ICH on the initial interpretation, all reports of the selected cases were analysed by a proprietary NLP tool (RepScheme, NLP tool). The NLP tool was configured using a sample of 1091 clinically-acquired reports, extraneous to the selected cases, that were manually classified as positive/negative for ICH by report. The CT imaging data corresponding to the reports that were classified as negative by NLP (“negative-by-report” cases) were analysed by the AI software for ICH detection.

#### Discrepancy review

The cases with discrepancies—negative-by-report but positive-by-AI—were reviewed by a board-certified neuroradiologist. The reviewing neuroradiologist determined if the AI output correctly identified a missed ICH. The medical records of the patients with missed ICH were reviewed to identify any associated adverse outcomes.

### Prospective validation

The ICH detection software prospectively analysed all NCCT scans of the head for a 6-month trial period. The CT scans were interpreted by either consultant neuroradiologists, consultant radiologists, or senior radiology registrars. A board-certified neuroradiologist then performed a second read on cases from November 2019 to December 2019. This formed the ground truth for the validation of the ICH detection software, by which sensitivity, specificity, positive predictive value (PPV) and negative predictive value (NPV) were calculated. The confidence intervals for all statistics are exact binomial confidence intervals constructed using the method of Clopper and Pearson^13^.

The turn-around-time (TAT) was measured as the time from the completion of the scan to the report being finalised on the radiology information system (RIS). The post-implementation TAT (November 2019 to December 2019) was compared with a pre-implementation TAT (July 2019 to August 2019). A p-value of less than 0.05 was considered statistically significant.

A short survey was distributed to the radiologists asking their usage of Aidoc as percentage of their reporting time: 0%, 25%, 50%, 75% and 100%. The radiologists were also asked to provide general feedback on their experience using Aidoc.

### Ethics approval

This study was performed in line with the principles of the Declaration of Helsinki. Approval was granted by The Alfred Hospital Human Research and Ethics Committee (22nd Aug 2019, Project No. 519/19). The Ethics Committee considered that study as low-risk and the requirement for patient informed consent was waived.

## Results

### Retrospective peer review

**Figure 2:**
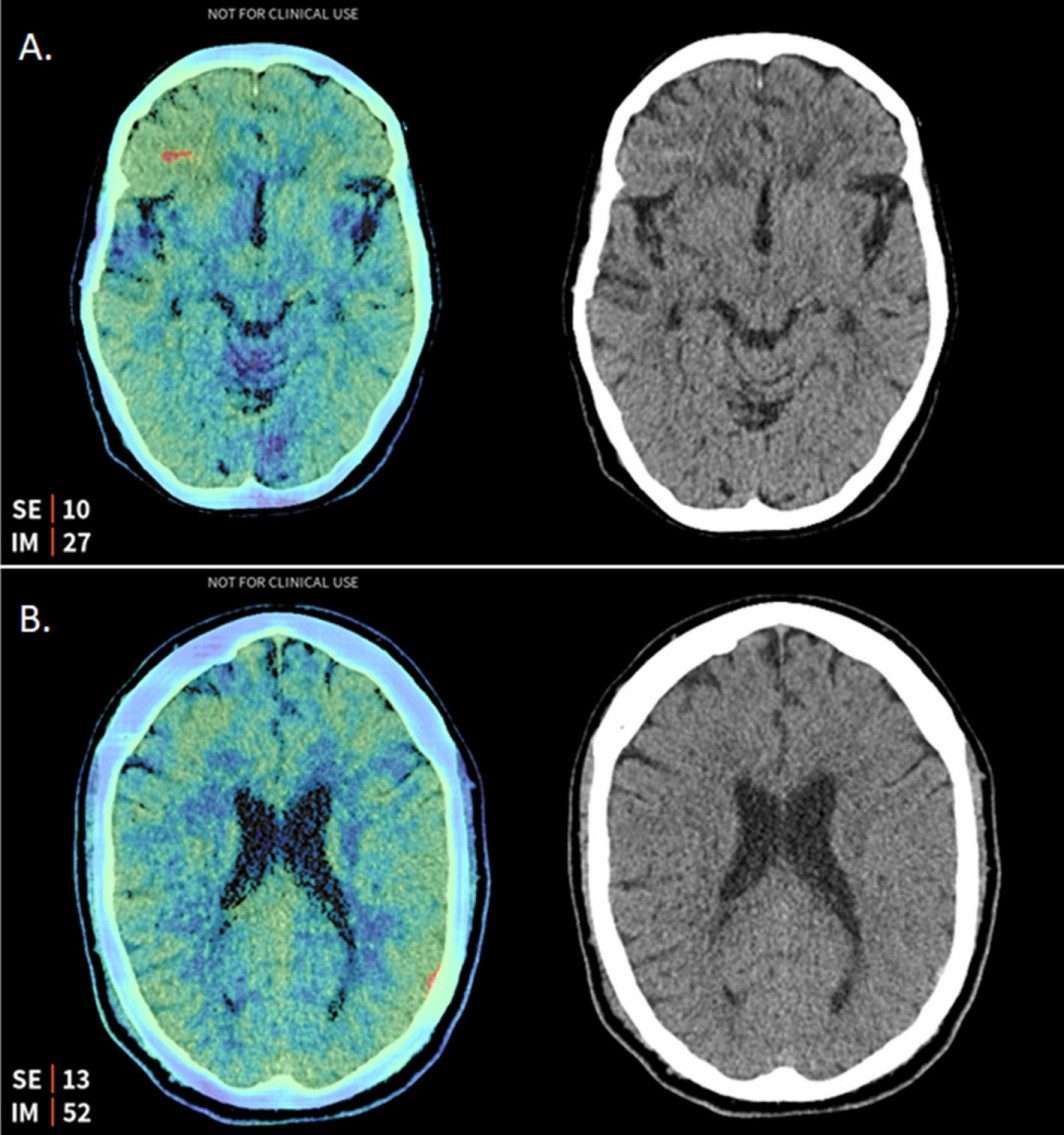
Two missed ICH detected by Aidoc. The key slices [right] and the saliency map generated by Aidoc [left] of the missed ICH identified by the audit; (**A**) a subarachnoid haemorrhage in the inferior right frontal lobe; and (**B**) a trace of extra-axial blood in the left parietal region.

The dataset for the retrospective review included 2916 head CT scans with corresponding reports. 20 negative-by-report CT scans were flagged by Aidoc. Ten of the 20 flags were false-positives caused by artefacts (n = 6), calcification (n = 2), falx cerebri (n = 1) or the straight sinus (n = 1). One flag was equivocal as the hyperdense focus could relate to either a small contusion or a hyperdense lesion. Nine were deemed ICH-positive by the reviewing neuroradiologist; two were stealth reformats, with the haemorrhages described in a separate report; three were haemorrhages in keeping with expected postoperative appearances. Of the remaining four ICH-positive scans, two had follow-up imaging where the haemorrhage had resolved, and two had no follow-up imaging. One of the misses was a small area of subarachnoid haemorrhage, and the other was a very small extra-axial haemorrhage (Fig. 2).

Excluding the flagged scans that were stealth and postoperative imaging, the emergency department were contacted with the findings and addenda were issued to all reports that missed an ICH. The emergency department arranged contact with the two patients which did not have any follow-up imaging. Both patients exhibited no long-term sequelae.

### Prospective validation

For the prospective validation, a total of 1446 head CT scans were analysed by Aidoc for the presence of ICH. The reviewing neuroradiologist identified 212 of these as ICH-positive; a prevalence of 14.7%. The prevalence of ICH in the emergency cohort was 6.3% (56/884), inpatient 30.3% (141/456) and outpatient 13.4% (13/97).

Aidoc flagged 220 as ICH-positive, of which 180 (81.81%) were true-positive. Out of the 1226 scans that were not flagged by Aidoc, 30 (2.5%) were false-negatives.

The diagnostic accuracy of the software for all cases was as follows: sensitivity 85.7% (95% CI 80.3–90.2%); specificity 96.8% (95% CI 95.6–97.6%); PPV 81.8% (95% CI 76.8–86.0%), NPV 97.6% (95% CI 96.6–98.2%). The diagnostic accuracy stratified by location is shown in Table 1. The specificity and NPV were consistent between the different locations. The sensitivity and PPV were highest in the inpatient cohort (90.1% and 93.4%), followed by emergency (82.1% and 68.8%), then outpatients (53.8% and 53.8%).

**Table 1:** Confusion matrix with calculated sensitivity, specificity, positive predictive value (PPV) and negative predictive value (NPV) of Aidoc ICH in emergency, inpatient, and outpatient CT head scans.

	Flagged	Not flagged	
**Emergency**			
ICH-Positive	46	10	
ICH-Negative	25	803	
**Inpatient**			
ICH-Positive	127	14	
ICH-Negative	9	465	
**Outpatient**			
ICH-Positive	7	6	
ICH-Negative	6	78	

	Emergency	Inpatient	Outpatient
Sensitivity (95% CI)	82.1% (69.6–91.1%)	90.1% (83.9–94.5%)	53.8% (25.1–80.8%)
Specificity (95% CI)	97.0% (95.6–98.0%)	97.2% (94.8–98.7%)	92.9% (85.1–97.3%)
PPV (95% CI)	64.8% (55.1–73.4%)	93.4% (88.1–96.4%)	53.8% (31.7–74.6%)
NPV (95% CI)	98.8% (97.9–99.3%)	95.7% (93.2–97.4%)	92.9% (87.8–95.9%)

The false-positive scans had a single or multiple feature(s) that were incorrectly identified as an ICH. The majority of these features were normal or calcified falx cerebri (9/40, 22.5%), followed by artefacts (7/40, 17.5%), postoperative dural thickening (6/40, 15.0%), meningioma (4/40, 10%), normal or calcified choroid (2/40, 5.0%), vessels (2/40, 5.0%), other calcification (2/40, 5.0%), developmental venous anomaly (1/40, 2.5%), cavernoma (1/40, 2.5%), encephalomalacia (1/40, 2.5%), hyperdense tumour (1/40, 2.5%), colloid cyst (1/40, 2.5%), dural venous sinus (1/40, 2.5%), pannus at C1-C2 (1/40, 2.5%), tentorium (1/40, 2.5%).

Nineteen of the 30 false-negatives were subacute ICH and 11 were acute ICH. The majority were subdural haemorrhages (12/30, 40%), followed by subarachnoid haemorrhages (6/30, 20%), post-operative extra-axial haemorrhages (4/30, 13%), intraparenchymal haemorrhages (3/30, 10%), other extra-axial haemorrhages (3/30, 10%) and basal ganglia haemorrhages (2/30, 7%). The inpatient and outpatient cohorts had a higher rate of subacute misses (10/13 and 4/6, respectively). Six acute and five subacute ICH were missed in the emergency cohort.

### Turn-around-time (TAT)

The turn-around-time (TAT) for the post-implementation dataset described above was compared with a pre-implementation dataset, which included 1628 head CT scans; 1469 (90.2%) were negative for ICH and 159 (9.8%) were positive for ICH. Both datasets had a similar proportion of emergency, inpatient, outpatient cases. The inpatient cohort had the highest incidence of ICH (18% pre, 31% post), followed by outpatients (9% pre, 13% post) then emergency (5% pre, 6% post).

For all ICH-negative scans, the mean pre-implementation TAT was 90.9 (SD 279.8) minutes, and the mean post-implementation TAT was 133.2 (SD 442.9) minutes. For all ICH-positive scans, the mean pre-implementation TAT was 66.7 (SD 41.5) minutes, and the post-implementation TAT was 80.0 (SD 54.25) minutes. The TAT stratified by emergency, inpatient and outpatient cases is shown in Table 2.

**Table 2:** Turn-Around-Time (TAT) for the pre- and post-implementation datasets stratified by patient location: emergency, inpatient and outpatient.

	All		Emergency		Inpatient		Outpatient	
	Negative	Positive	Negative	Positive	Negative	Positive	Negative	Positive
**Pre-implementation**								
No. of scans (n)	1469	159	914	53	433	94	122	12
Average TAT (SD, min)	90.9 (279.7)	66.7 (41.5)	55.7 (36.0)	71.8 (40.1)	72.2 (92.1)	63.5 (43.3)	421.0 (888.2)	69.7 (32.5)
**Post-implementation**								
No. of scans (n)	1234	212	827	57	323	142	84	13
Average TAT (SD, min)	133.2 (442.9)	80.0 (64.4)	65.4 (39.0)	68.1 (32.8)	80.0 (91.1)	86.1 (73.6)	1005.6 (1428.3)	59.8 (25.1)

There was a decrease in TAT for ICH-positive scans in the emergency and outpatient cohorts by 3.7 min (− 5.1%) and 9.9 min (− 14.2%), respectively. There was an increase in TAT for ICH-positive scans in the inpatient by 22.6 min (35.6%). The difference in TAT for all cases and the inpatient cases were statistically significant (*P* = 0.017 and *P* = 0.003). The difference in TAT for the emergency and outpatient cohorts were not statistically significant (*P* = 0.59 and *P* = 0.07).

Out of 49 consultant radiologists and registrars, 26 responded to the survey. Three radiologists used Aidoc 100% of their reporting time; three 75%; four 50%; seven 25% and nine 0%.

## Discussion

The ICH-detection module of the TGA-approved AI software, Aidoc, was assessed at one of Australia’s busiest trauma centres. Aidoc was used “off-label” to perform a retrospective audit of all non-contrast CT scans of the head to identify the institution’s miss-rate prior to the implementation of the Aidoc into the clinical workflow. The retrospective audit of 2916 scans identified two cases (0.07%) where a small ICH was not documented in the initial report and the patient had no follow up imaging. Despite the audit capturing only a month’s worth of data, the institution’s miss-rate was orders of magnitude lower than that reported in previous studies^14^. However, with the institution’s yearly average of 20,000 head CT scans, the miss-rate of approximately 1 in 1400 would lead to 13–14 missed ICH annually. This indicates the value of enhanced detection when using AI in time-critical indications such as ICH, and can serve as a QA second-read system.

The sensitivity and specificity of the AI showed comparable results to that reported in literature (4, 7–9), indicating generalisability in an Australian clinical context. The majority of false-positives were caused by calcifications and structures, such as the falx cerebri, that share similar density values as ICH^15^. A PPV of 82% equated to 1–2 cases in 10 that were incorrectly prioritised. As the dashboard accumulated these false-positives, the reporting radiologists were at risk of alert fatigue. This was exacerbated by the majority of flagged CT scans arising from the inpatient cohort, which were often follow-up imaging of previously documented haemorrhages. However, the issue was communicated with the Aidoc team who then deployed a feature that would display a “follow-up” label on flagged CT scans if the patient had a previously flagged scan within 7 days. The effect this had on reducing alert fatigue needs to be further investigated.

Interestingly, the outpatient cohort had a higher prevalence of ICH than the emergency cohort, yet the diagnostic accuracy of Aidoc was the lowest in the outpatient population. This study did not conduct a comprehensive failure mode analysis; however, the reduction in the sensitivity and PPV when analysing outpatient scans suggests that there is impaired performance when detecting subacute haemorrhages in the outpatient population. This implies that there is a systematic difference in not just the prevalence but the actual appearance or content of the outpatient CT scans—perhaps scanner based or pathology based i.e. smaller or non-hyperdense bleeds.

The key metric of this study that demonstrates Aidoc’s triaging performance was the turn-around-time (TAT). A decrease in TAT would indicate a timelier diagnosis and subsequent treatment, which can drastically improve patient outcomes. Interestingly, there was a statistically significant increase in the overall TAT for ICH-positive cases between the pre- and post-implementation groups. The TAT for the ED and OP ICH-positive cases was reduced, although it was not statistically significant. A major factor to consider is that the institution has a dedicated reporting room in ED allowing emergency physicians to inform the reporting radiologists which patients to prioritise based on the initial presentation. Additionally, communication between radiographers and radiologists assists with the prioritisation of urgent cases. This operational efficiency is depicted in the short TAT pre-implementation, causing any improvements from the use of AI to be marginal. Additionally, using TAT as a surrogate for the time taken for a clinical decision to be made has several drawbacks. For example, the reporting radiologist can communicate significant findings with the ED physicians before the report has been finalised on RIS. Hence, further refinement of this metric is required.

A limitation of this study was the departmental usage of Aidoc. From a convenience sample of 26 consultant radiologists, most only partially incorporated (25–75% of reporting time) Aidoc into their reporting workflow, and some did not use it at all. The collective opinion from the radiologists was that the ICH module represents a solution for a problem that the department does not currently have. They stated that the centre scans too many patients with ICH, many of which are repeat inpatient scans. Furthermore, as the software was implemented as a trial, there was limited integration allowed with the department’s PACS/RIS, which resulted in the Aidoc widget being installed on separate PCs in the reporting rooms, instead of each radiologist’s workstation. This added an element of inconvenience and resulted in the radiologists using Aidoc as a ‘second-read’ once they had reviewed the scan. Features such as worklist prioritisation and context matching were requested by the radiologists to improve their workflow but were not achievable with the institution’s PACS/RIS. These limitations led to a sub-optimal use of Aidoc throughout the department, which could be considered as one of the reasons why there was no significant impact on the TAT.

Currently, our institution’s imaging and reporting operations have a relatively short reporting time and low miss-rate. By performing a retrospective audit in addition to a standard validation, this study presents a framework to review the function and use of AI software, and also highlighting the challenges of tailoring AI diagnostic solutions to departmental needs. Further research into the changes in reporting time pre- and post-implementation is required to assess the triaging capabilities and impact of the AI solution. Any improvements, as a result of AI implementation, would provide further evidence to support the use of AI technology in radiology to increase radiologists’ efficiency and improve patient care.

## Supplementary Information

Supplementary Information 1.

Supplementary Information 2.

Supplementary Information 3.

## Data Availability

All data generated or analysed during this study are included in this published article [and its supplementary information files].
